# Mode-assisted Silicon Integrated Interferometric Optical Gyroscope

**DOI:** 10.1038/s41598-019-49380-x

**Published:** 2019-09-10

**Authors:** Beibei Wu, Yu Yu, Xinliang Zhang

**Affiliations:** Wuhan National Laboratory for Optoelectronics, Huazhong University of Science and Technology, Wuhan, 430074 China

**Keywords:** Integrated optics, Silicon photonics

## Abstract

The increasing demands in consumer electronics markets have promoted the development of chip-scale optical gyroscopes. In this study, a mode-assisted on-chip silicon-on-insulator interferometric optical gyroscope is proposed and assessed. The proposed gyroscope uses two different spatial modes propagating oppositely in the sensing waveguide coil to form a fixed phase difference that ensures the system operating at the best sensitive point. Compared with conventional schemes, it avoids the phase modulator and the circulator, which are not easy to be integrated in the same platform. The simulated results show that the detectable angular rate reaches 0.64 deg/s with a footprint of 3.85 × 10^−3^ m^2^. The experimental results validate the realization of the highly sensitive phase bias of the fabricated device.

## Introduction

The optical gyroscope, based on the Sagnac effect^1^, is a distinguished technology for angular velocity sensing. It has the advantages of high precision, good mechanical reliability and immunity to the electromagnetic interference. The well-established fiber optic gyroscopes (FOGs) and ring laser gyroscopes (RLGs) have been routinely applied in the spacecraft, satellite, airplane, missile, and submarine fields^2^. In recent years, the emerging applications in cellphones, automotives and robotics have urgently increased the demands on lightweight and low-cost optical gyroscopes. To this end, considerable configurations of integrated optical gyroscopes have been proposed. These chip-scale optical gyroscopes were based on various kinds of platforms such as the indium phosphorus (InP)^3^, silica^4^, silicon nitride^5–7^ and calcium fluoride^8^. Especially, the silicon-on-insulator (SOI) platform is now becoming a promising candidate due to the advantages of compactness, high density, low cost and compatibility with the complementary metal-oxide-semiconductor (CMOS) fabrication technology. Furthermore, it has the potential to realize the hybrid photonic-electronics integration on a single chip for prospective commercialization. The optical gyroscopes based on the SOI platform were subsequently investigated including both the interferometric and resonant schemes^9–11^.

**Figure 1:**
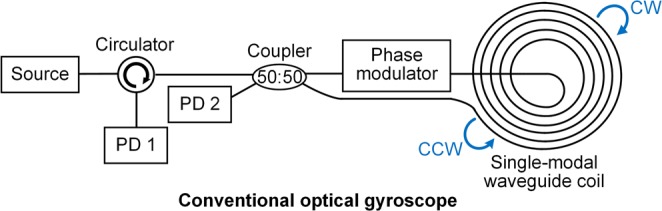
Schematic of conventional optical gyroscope. PD: photodetector.

The configuration of conventional interferometric optical gyroscopes is shown in Fig. 1. Light from the source is divided by 50:50 coupler to form clockwise (CW) and counter-clockwise (CCW) beams in the single-mode waveguide coil. When the gyroscope rotates perpendicularly to the plane, the counter-propagating beams undergo different phase shifts, which are proportional to the rotation velocity. After interfering in the 50:50 coupler, the phase shifts are converted to intensity information and detected by the photodetectors (PDs) for measurement. The phase modulator (PM), which is used to adjust the optimal sensitive operation bias of the gyroscope, is an indispensable part for conventional interferometric rotation sensing. The circulator, which is used to separate the input and output beams^12^, is difficult to fabricate in the integrated scheme actually. Hence, the circulator was normally replaced by a 3 dB coupler, which led to extra loss and reflection light to light source, in the integrated scheme^13^. In previous studies, an integrated solution based on the silicon substrate was proposed by employing hybrid integration technology^5–7^. The ultralow loss waveguide coil was fabricated on the silicon nitride platform, whereas the PM was fabricated on the III-V platform and bonded to the silicon layer. However, the fabrication process was difficult and expensive, and it might not be applicable for massive production. Therefore, it is significant to remove the PM and circulator in the integrated gyroscope scheme while the highly sensitive detection is still available.

Commonly, the mode-division multiplexing technology uses multiple eigenmodes to increase the link capacity in the communication systems^14,15^. Few studies have showed its potential in the rotation measurement field. In this paper, we propose a mode-assisted on-chip SOI interferometric optical gyroscope by using two different spatial modes counter propagating in the waveguide sensing coil. The counter-propagating modes induce a fixed phase difference that achieves a highly sensitive detection. Compared with conventional schemes, the proposed one avoids the PM and circulator, thus significantly reducing the complexity and fabrication difficulty. The configuration of the scheme, theoretical analysis, numerical modeling, and stationary experimental results are demonstrated in the following section.

## Results and Discussion

**Figure 2:**
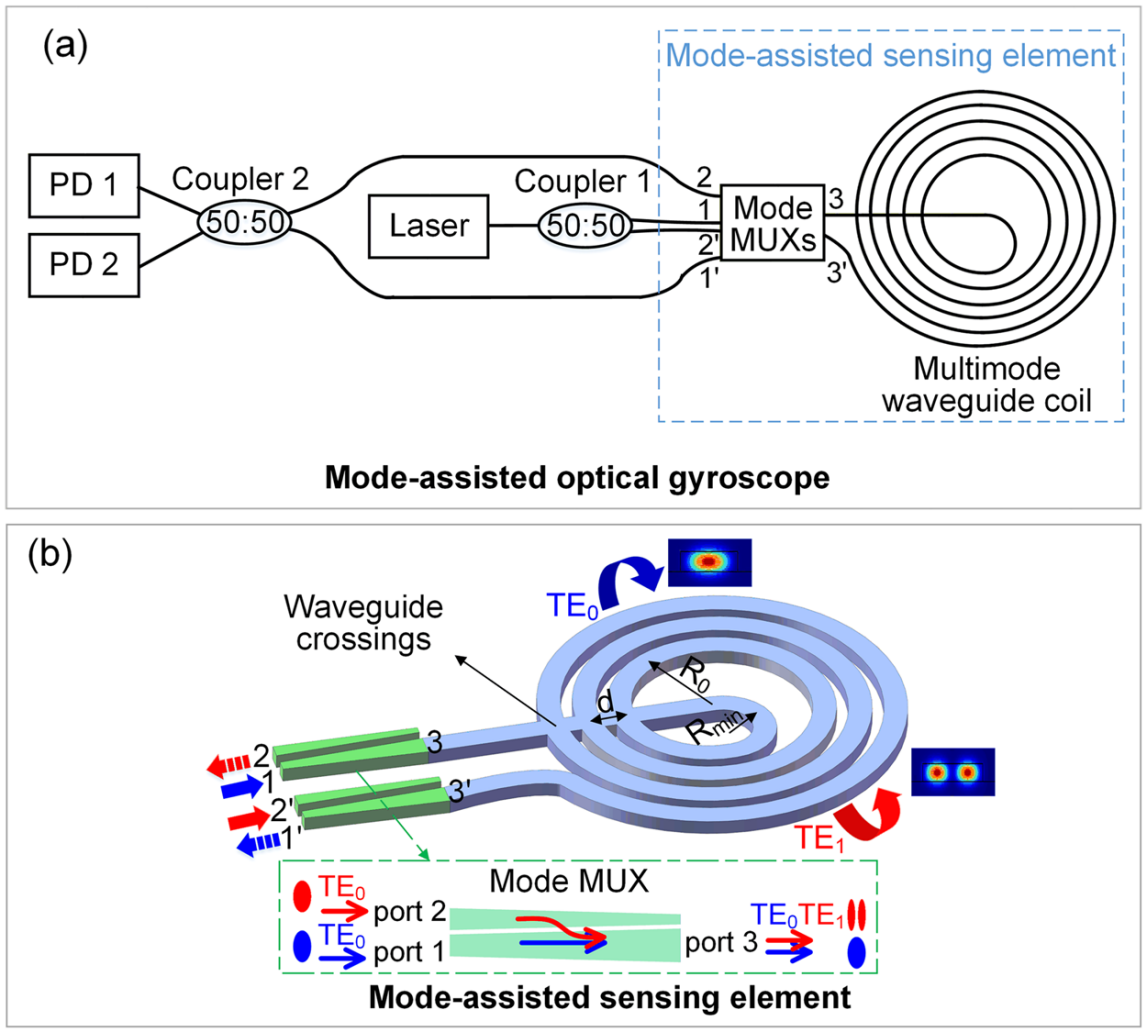
(**a**) Schematic of proposed mode-assisted optical gyroscope. (**b**) Configuration of mode-assisted sensing element. The inset in the green dashed box is the schematic of mode MUX.

The schematic of the proposed mode-assisted integrated gyroscope is illustrated in Fig. 2(a). It consists of a laser source, two 50:50 couplers, two PDs and a sensing element, which includes two identical mode multiplexers (MUXs) and a multimode waveguide coil. All the passive optical components are designed on the SOI platform. The mode MUX, whose 3 ports are marked as port 1, 2, and 3, is composed of the counter-tapered directional couplers based on the mode evolution theory^16^, as shown in the inset of Fig. 2(b). In the mode MUX, the fundamental transverse electric polarized mode (TE_0_) injected from port 1 will be transferred to port 3 and keep unchanged, while TE_0_ mode injected from port 2 will be converted to first-order transverse electric polarized mode (TE_1_) at port 3. The ports of another identical mode MUX are correspondingly marked as port 1′, 2′, and 3′. The multimode waveguide coil consists of strip waveguides and waveguide crossings. The waveguide spacing *d* depends on the size of waveguide crossing. The innermost waveguide radius is expressed as *R*_0_. The minimum waveguide radius *R*_*min*_ depends on *R*_0_ and *d*. The waveguide crossings in the multimode waveguide coil were designed for both TE_0_ and TE_1_ modes in our previous work^17^. The insertion losses of the dual-mode waveguide crossing are 0.04 dB and 0.08 dB for TE_0_ and TE_1_ modes in theory, respectively. Its footprint is 33.7 μm × 33.7 μm. The ports of the two mode MUXs are connected with multimode waveguide coil and two 50:50 couplers, as shown in Fig. 2(a,b).

In the proposed mode-assisted optical gyroscope, a laser source is utilized, since the phase difference between different modes is highly dependent on the source wavelength. The light from the source is divided by the coupler 1. The two divided beams are injected to the mode MUXs, at port 1 and port 2′, to form TE_0_ and TE_1_ modes at port 3 and port 3′, respectively. The undesired TE_1_ mode converted at port 3 and TE_0_ mode converted at port 3′ as the crosstalk could be filtered when reaching the mode MUXs again, and would not be detected by the PDs. In the coil part, the TE_1_ and TE_0_ modes with CCW and CW propagations experience the rotation, respectively, as the counter-propagating beams will undergo different rotation-dependent phase shifts. Then, the TE_1_ mode reaches port 3 and is converted back to TE_0_ mode at port 2, whereas the unconverted TE_0_ mode reaches port 3′ and maintains unchanged at port 1′. Finally, the two beams interfere at the coupler 2, converting the rotation-dependent phase shifts into intensity information to be detected by PDs. To simplify the scheme, the laser and the PDs here are actually input and output grating couplers, coupling the light to the outside fiber.

In the conventional schemes, an optimized sensitivity was realized by applying a time-dependent phase modulation to the CW and CCW beams to form a π/2 phase difference, and the interference plot showed maximum slope^2^. In the proposed scheme, the two spatial modes propagating in the same waveguide coil induce a fixed phase difference, expressed as *ϕ*_*m*_, due to different effective indexes. On the other hand, the rotation induces an additional phase difference between counter-propagating beams, expressed as *ϕ*_*r*_. The mode-dependent phase difference *ϕ*_*m*_ is to adjust the operation bias of the gyroscope while the rotation-dependent phase difference *ϕ*_*r*_ is the target to be measured. When *ϕ*_*m*_ is *m*·π/2, where *m* is an odd, the gyroscope realizes highly sensitive detection. Compared with conventional schemes, the mode-assisted integrated interferometric optical gyroscope avoids utilization of PM and circulator.

However, no studies on dual-mode optical gyroscope have been proposed so far. The theoretical analysis and numerical model should be reinvestigated. Based on the Sagnac effect and in analogy with single-mode case^18^, the velocities of the two modes in the CW and CCW directions in the SOI waveguide measured by a stationary observer are given in the following with a first-order approximation of Ω*R*, considering the CW direction is positive,

1 $${u}_{CW}=\frac{c}{nef{f}_{0}}+\Omega R{\zeta }_{0}$$

2 $${u}_{CCW}=\frac{c}{nef{f}_{1}}-\Omega R{\zeta }_{1}$$

where Ω is the angular velocity of the rotating device, *R* is the radius of the circled waveguide area, *neff*_0_ is the effective index of TE_0_ mode, *neff*_1_ is the effective index of TE_1_ mode, *c* is the velocity of light in vacuum, *ζ*_*j*_ is the Fresnel-Fizeau Drag coefficient, and *ζ*_*j*_ = 1 − (1/*neff*_*j*_)^2^ (*j* = 0, 1). The accumulated phases of the CW and CCW beams are given by,

3 $${{\rm{\varphi }}}_{CW}={\beta }_{0}\cdot \frac{L}{{u}_{CW}}\frac{c}{nef{f}_{0}}$$

4 $${{\rm{\varphi }}}_{CCW}={\beta }_{1}\cdot \frac{L}{{u}_{CCW}}\frac{c}{nef{f}_{1}}$$

where *β*_0_ is the propagating constant of TE_0_ mode, *β*_1_ is the propagating constant of TE_1_ mode, and *L* is the total length of the encircled waveguide. The detected total phase difference of the multi-modal gyroscope can be deduced into the following expression with a first-order approximation of Ω*R*,

5 $$\Delta {{\rm{\varphi }}}_{total}=L(nef{f}_{0}-nef{f}_{1})\frac{2\pi }{\lambda }+\frac{8\pi {A}_{sum}}{c\lambda }\Omega ={\varphi }_{m}+{\varphi }_{r}$$

where *λ* is the operating wavelength, and *A*_*sum*_ is the total area of the encircled waveguide, including the coiled areas. The second item of Eq. (5) is proportional to Ω, and it shows that the rotation-induced phase shift of multi-modal gyroscope equals to that of the single-mode one. The Sagnac effect similarly applies to multi-modal gyroscope. The detectable angular rate of the proposed gyroscope is dependent on the slope of the output interference plot. *T*_*s*_ is the normalized output interference plot of the device, and it changes with phase shift. *ϕ* denotes the phase shift. *∂T*_*s*_*/∂ϕ* is its first derivative, i.e. the slope of the output response. The detectable angular rate can be deduced as below^18^, and it is given by,

6 $${{\rm{\delta }}\Omega }_{min}=\frac{{\delta }_{i}c\lambda }{8\pi {A}_{sum}{i}_{D}{(\partial {T}_{s}/\partial \varphi )}_{max}}$$

where *i*_*D*_ is the photodiode current, and *δ*_*i*_ represents the standard deviation of the photocurrent, considering shot noise, thermal noise, and laser noise in the system.

**Figure 3:**
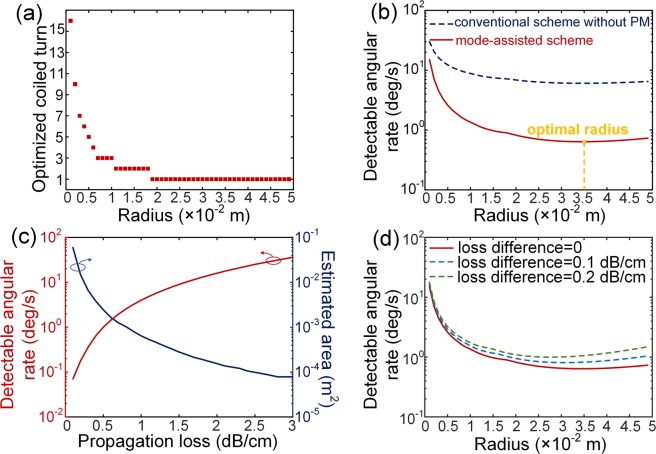
Simulated results of (**a**) optimized coiled turn numbers, (**b**) detectable angular rate, (**c**) optimal detectable angular rates and estimated optimal areas, and (**d**) detectable angular rate degradation induced by loss imbalance.

For simulation, the waveguide propagation loss is assumed to be 0.4 dB/cm^19^, and the waveguide crossing loss is assumed to be 0.08 dB/crossing for both modes. The parameters of *δ*_*i*_ used in simulation are with the typical values as follows: the fundamental electric charge *q* =1.6 × 10^−19^ C, Boltzman’s constant *k*_*B*_ = 1.38 × 10^−23^ J/K, temperature *T* = 298.15 K, photodetector load resistance *R*_*L*_ = 100 Ω, relative intensity noise *RIN* = −160 dB/Hz^20^, and bandwidth *B* = 10 Hz. The photodiode current *i*_*D*_ is 1 mA. The waveguide spacing *d* is 33.7 μm. The minimum waveguide radius *R*_*min*_ is (*R*_0_ + *d*/2)/2. The coiled turn numbers are scanned for each innermost radius *R*_0_, and Fig. 3(a) shows the optimal coiled turn numbers for detectable angular rate corresponding to each radius *R*_0_. It indicates that larger waveguide radius has less optimal coiled turns, because the larger device has larger propagation loss. In Fig. 3(b), the red solid line represents the detectable angular rate of the proposed mode-assisted scheme. It can be found that there is an optimal *R*_0_ and corresponding detectable angular rate under one propagation loss. The blue dashed line represents the detectable angular rate of conventional scheme without PM. It can be found that the detectable angular rate of mode-assisted gyroscope scheme shows up to 10 times enhancement than that of conventional one without PM. A simulated detectable angular rate of 0.64 deg/s can be obtained with a footprint around 3.85 × 10^−3^ m^2^.

The relation of the optimal detectable angular rate and corresponding optimal area for each propagation loss is illustrated in Fig. 3(c). The optimal detectable angular rate decreases while its corresponding area increases, with decreasing propagation loss. The results indicate that better performance can be expected when smaller propagation loss and larger area are available.

In practical case, the propagation loss of higher order mode is larger than that of fundamental one in a silicon waveguide. In Fig. 3(d), the red solid line shows the detectable angular rate with a propagation loss of 0.4 dB/cm for both modes. The dashed lines show the detectable angular rate when the loss differences are assumed to be 0.1 and 0.2 dB/cm for blue and green lines respectively as an example. The detectable angular rate with loss difference is getting worse because of the incomplete interference between the two counter beams with larger loss difference.

**Figure 4:**
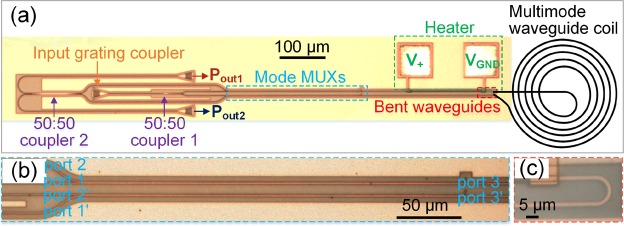
(**a**) Metalloscope photo of the fabricated device. (**b**) Zoom-in photo of mode MUXs. (**c**) Zoom-in photo of ultra-compact bent waveguides.

The device is fabricated, and Fig. 4(a) is the metalloscope photo of the fabricated device, which includes three grating couplers, two 50:50 couplers, two mode MUXs, a metal pad heater, and two ultra-compact bent waveguides for connecting^21^. The black line represents the multimode waveguide coil, which is not included in the fabricated device. Figure 4(b,c) are the zoom-in photos of the MUXs and bent waveguides respectively.

**Figure 5:**
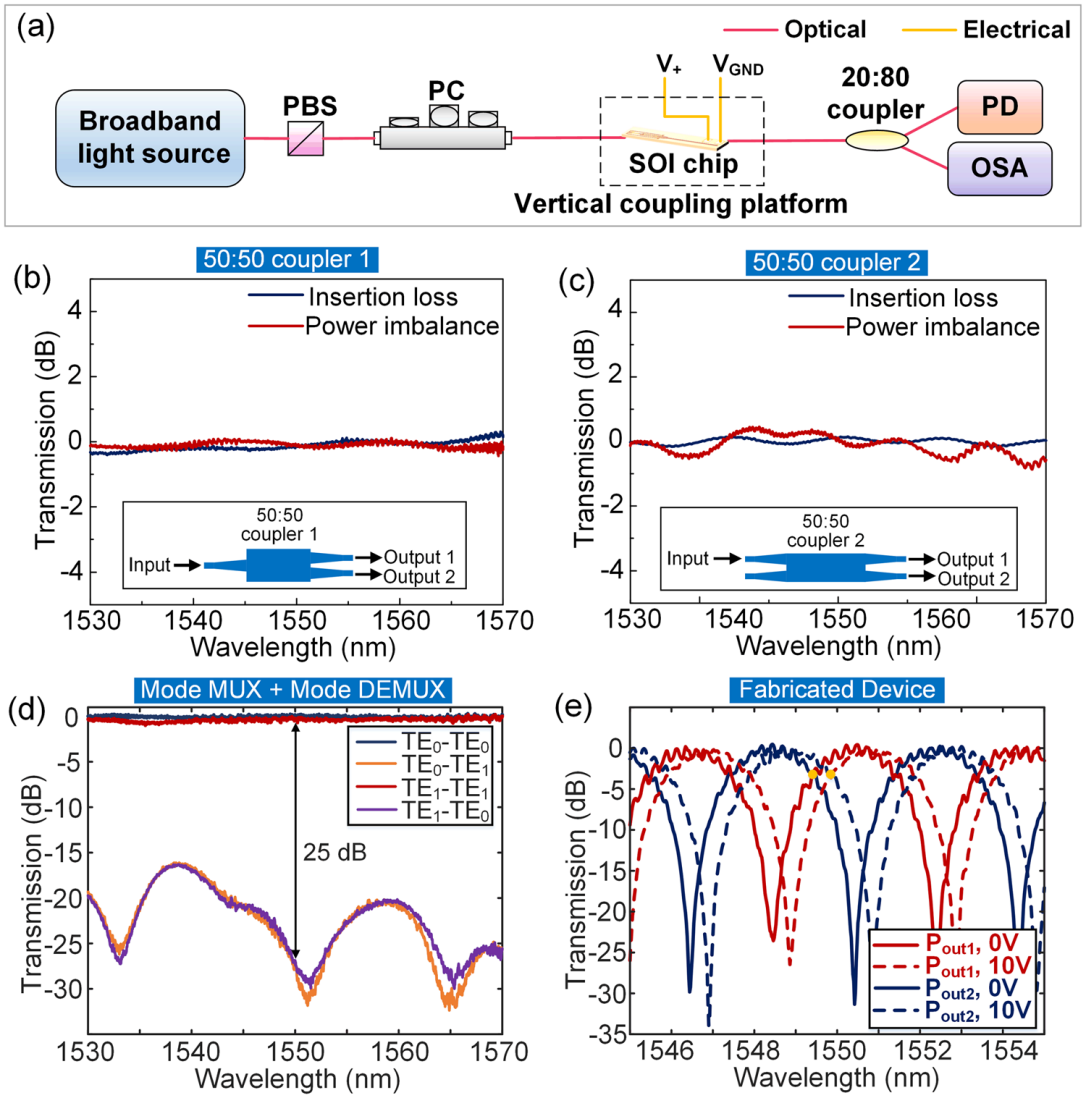
(**a**) Experimental setup. PBS: polarization beam splitter; PC: polarization controller; OSA: optical spectrum analyzer. Measured spectra of the reference (**b**) 50:50 coupler 1 and (**c**) 50:50 coupler 2. (**d**) Measured spectra of the reference mode MUXs. (**e**) Measured spectra of the fabricated device.

Fig. 5(a) presents the experimental setup. The beam from broadband light source is launched to the polarization beam splitter (PBS) and polarization controller (PC) to obtain a linear polarized beam aligned with the on-chip polarized state. The input and output coupling fibers are fixed on the fiber vertical coupling platform. Then, the linear polarized beam is injected to the chip via an input coupling fiber and an input grating coupler. The outputs are detected by a PD and an optical spectrum analyzer (OSA) via an output grating coupler, an output coupling fiber, and a 20:80 coupler. The PD is used to monitor the output power to be the highest, and the OSA is used to measure the output spectral response of the test device. The “V_+_” and “V_GND_” represent the positive electrode and ground of the metal pad, respectively. A voltage source is applied on the metal pads to heat the silicon waveguide. The spectral response of the fabricated device is determined by a combination of coupling coefficient, loop length, mode conversion efficiency, and the existence of multi-modal behavior^22,23^. Therefore, the characterization results of the fabricated 50:50 couplers and the mode MUX are necessary. By subtracting the grating coupler losses, the measured spectra of reference 50:50 couplers, which are 1 × 2 and 2 × 2 multimode interference couplers respectively, are shown in Fig. 5(b,c). The insets show the schematics of the couplers with input and output ports. The power imbalance indicates the measured power difference between two output ports. The measured spectra of reference mode MUX and de-multiplexer (DEMUX), including the transmission and crosstalk of TE_0_ and TE_1_ links, are shown in Fig. 5(d). The stationary experiments without rotation are demonstrated in the following. Figure 5(e) shows the measured transmission spectra of the fabricated device in a logarithmic scale. The red and blue solid lines represent the output spectra with no voltage applied on the heater. When no rotation is applied, the phase difference only depends on the wavelength. The output spectra, which are the interference responses of two counter-propagating beams, are sinusoidal responses in a linear scale. The operating wavelength should be set at the crossing point of the two output spectra (shown as the orange dots), since it is the maximum slope of the normalized spectra. When the wavelength is fixed at that point, the rotation-induced additional phase difference has the maximum response, showing a highly sensitive detection for rotation measurement. When the multimode waveguide coil is added, the maximum slope of the normalized spectra can be adjusted accordingly. Moreover, the red and blue dashed lines represent the shift of output spectra when a voltage of 10 V is applied on the heater. The effective indexes of both modes will change by heating the silicon waveguide, according to the large thermo-optic coefficient of the silicon^24^. The effective index changing rates of TE_0_ and TE_1_ modes are slightly different. Thus, to heat the dual-mode waveguide, where both the TE_0_ and TE_1_ modes propagate, adjusts the highly sensitive operating point of the device slightly. This adjustment can be utilized to partly compensate the fabrication error and mismatch.

**Figure 6:**
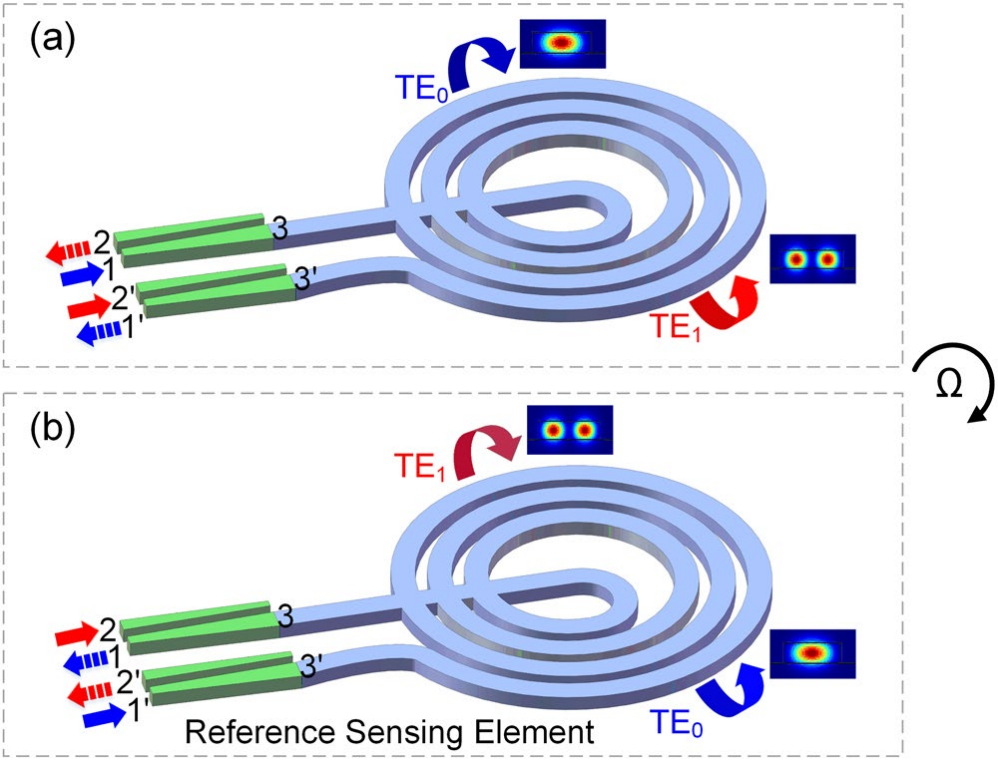
(**a**) Above mentioned sensing element. (**b**) Reference sensing element.

The stability of the system should be considered, since the described gyroscope has broken the reciprocity by employing two different modes, which renders the system insensitive to the thermal or mechanical fluctuations. The effective indexes of TE_0_ and TE_1_ modes change differently with temperature. After propagating in the long-length sensing waveguides, the modes induce phase bias *ϕ*_*m*_, which will drift with unwanted fluctuations. The phase drift causes undesired signals for the rotation measurement. For the phase error accumulation due to multiple modes, the multimode FOG using symmetrical multimode coupler and low coherent light source was proposed to mitigate the errors^25^. However, the solution is incompatible with our on-chip scheme. Besides, the coherent backscattering errors and reflection errors of the mode MUX induced by laser source^26^ are existing in the integrated optical gyroscope. The reflections of on-chip mode MUX should be characterized here. In Fig. 5(d), the measured transmission spectra of the reference mode MUX and DEMUX shows low insertion losses for TE_0_ and TE_1_ links, indicating low reflections of the fabricated mode MUXs. Additional simulated results show that the reflections of TE_0_ mode to TE_0_ and TE_1_ modes are <−59 dB and <−54 dB while the reflections of TE_1_ mode to TE_0_ and TE_1_ modes are <−52 dB and <−36 dB from the mode MUX to the waveguide coil at 1550 nm. To eliminate the errors and drifts, the sensing element cooperating with an identical reference sensing element is proposed in our scheme, as shown in Fig. 6. The light from the laser source is injected into the sensing element and the reference sensing element simultaneously. In the reference sensing element, the TE_0_ and TE_1_ modes propagate with the opposite directions of that in the sensing element in Fig. 6(a). The modes propagate along the same optical path in two structures, experiencing identical phase shift and phase errors. However, the rotation-induced phase shifts are directional, and the detected rotation signals are reverse in two structures. Therefore, by subtracting the detected signals of the sensing element and reference sensing element, the errors can be eliminated and the rotation signals can be extracted. Furthermore, the mode MUX at the output port can filter the backscattering and reflection lights, which are without mode conversion, effectively reducing the reflection errors. More specifically, in the structure in Fig. 6(a), the TE_0_ and TE_1_ modes propagate in the waveguide coil CW and CCW. The total phase difference is calculated in Eq. (5), i.e. *Δϕ*_*total*_ = *ϕ*_*m*_ + *ϕ*_*r*_, considering a CW rotation. When the light launched into the reference sensing element from port 2 and port 1′, as shown in Fig. 6(b), the TE_0_ and TE_1_ modes propagate in the waveguide coil CCW and CW. The total phase difference in Eq. (5) is rewritten as *Δϕ*_*total*_ = *−*(*ϕ*_*m*_ − *ϕ*_*r*_). Any stochastic variation of the environment fluctuates *ϕ*_*m*_ rather than *ϕ*_*r*_. The output interfered intensity signals of the two structures in Fig. 6(a,b) contain common-mode signal *ϕ*_*m*_. The fluctuation-induced noise can be cancelled by subtracting the detected signals, and the rotation-induced phase shift *ϕ*_*r*_ can be extracted. It is worth noting that the fabrication inconformity of the two sensing elements will degrade the reciprocity of the gyroscope introducing extra noise in the system. The sensing element and the reference one are fabricated on the same die to reduce the fabrication inconformity. In addition, the phase bias drift can be compensated by controlling the metal pad heater dynamically for highly sensitive detection.

In summary, an on-chip silicon mode-assisted interferometric optical gyroscope using different modes propagating oppositely in a waveguide coil is proposed and investigated. This gyroscope avoids integrating the PM and circulator, and reduces the cost and fabrication difficulty. A simulated detectable angular rate reaches 0.64 deg/s with a footprint of 3.85 × 10^−3^ m^2^. The experimental results show the elementary functionality of the designed device. We believe that this scheme will accelerate the development in the integration of the optical gyroscope on a silicon chip.

## Method

### Simulation method

The simulated detectable angular rates of the proposed integrated interferometric optical gyroscope are numerically calculated by Matlab code for parameter optimizing.

### Device fabrication

The waveguides are fabricated on a 0.22 μm silicon top layer surrounded by silica cladding with a 2 μm silica box layer. The standard 248 nm deep ultraviolet (UV) lithography and the inductively coupled plasma (ICP) etching processes are used in the fabrication.
